# Development of chloroplast transformation and gene expression regulation technology in land plants

**DOI:** 10.3389/fpls.2022.1037038

**Published:** 2022-10-27

**Authors:** Yaqi An, Yue Wang, Xinwei Wang, Jianwei Xiao

**Affiliations:** ^1^ College of Biological Sciences and Biotechnology, Beijing Forestry University, Beijing, China; ^2^ Key Laboratory of Genetics and Breeding in Forest Trees and Ornamental Plants, Ministry of Education, Beijing Forestry University, Beijing, China; ^3^ The Tree and Ornamental Plant Breeding and Biotechnology Laboratory of National Forestry and Grassland Administration, Beijing Forestry University, Beijing, China; ^4^ College of Agriculture and Forestry, Hebei North University, Zhangjiakou, China

**Keywords:** chloroplast genome, gene expression, transformation, aptamer, dPPR, CRISPR/Cas

## Abstract

Chloroplasts in land plants have their own small circular DNA that is presumed to have originated from cyanobacteria-related endosymbionts, and the chloroplast genome is an attractive target to improve photosynthetic ability and crop yield. However, to date, most transgenic or genetic engineering technologies for plants are restricted to manipulations of the nuclear genome. In this review, we provide a comprehensive overview of chloroplast genetic engineering and regulation of gene expression from the perspective of history and biology, focusing on current and latest methods. In addition, we suggest techniques that may regulate the chloroplast gene expression at the transcriptional or post-transcriptional level.

## Introduction

Chloroplasts are photosynthetic organelles that exist in the cells of some protists and plants, which are critical for their functional integrity and viability. The genome (plastome or ptDNA) of chloroplasts is 120–180 kb in size and is characterized by highly polyploid, circular, double-stranded DNA. Chloroplast DNA encodes approximately 120 genes in land plants, most of which play essential roles in chloroplast development or photosynthesis. The development of chloroplast genetic engineering has lagged behind that of nuclear genetic engineering despite its advantages (Boynton et al., 1988)—for instance, homologous recombination (HR) is prevalent in chloroplasts; therefore it is not necessary to introduce extra double-stranded DNA for cutting. In addition, chloroplasts in land plants are usually maternally inherited, resulting in the natural biological limitation of transgenes through pollen escape. Finally, like prokaryotic gene expression, the design, construction, and regulation of metabolic pathways are likely manipulated by operons, which produce polycistronic transcripts in plastids. All these properties are very promising for future metabolic engineering.

Regulation of chloroplast gene expression through chloroplast genome engineering has been used to produce high-value industrial targets, improve photosynthetic capacity, biofortify food crops, *etc*. (Boynton et al., 1988). Over years of research, plastids, in general, and chloroplasts, in particular, have emerged as novel platforms for plant genetic engineering. The development and application of chloroplast genome engineering technology may lead to increases in the study of chloroplast gene functions, gene editing, gene expression regulation, and genome analysis. This review summarizes the methodology and new tools used in chloroplast transformation, discusses current restrictions and future prospects in chloroplast genome engineering, and also proposes possible applications of the new technologies in chloroplast gene expression.

## Development of chloroplast transformation technology

Chloroplast transformation provides a valuable alternative platform to generate transgenic plants. In seed plants, chloroplast transformation will face greater challenges because it always contains 1,000–2,000 copies of the chloroplast genome and approximately 100 chloroplasts per mature leaf mesophyll cell (Golczyk et al., 2014). Over the last several decades, the fundamental methods of introducing foreign DNA into chloroplasts have not significantly changed (Zienkiewicz et al., 2017; Kumar and Ling, 2021). Chloroplast transformation was once realized through biolistic delivery, polyether polyethylene glycol (PEG)-mediated delivery, or *Agrobacterium*-mediated delivery. These approaches require the regeneration of genetically modified progeny plants, which can be challenging and require lengthy procedures. Therefore, effective transformation protocols have been designed for a very limited range of plants to date (Ruf et al., 2021; Kumar and Ling, 2021). The traditional transformation techniques have important limitations—for example, PEG-mediated transformation requires the subsequent regeneration of stable plant lines from isolated protoplasts that have their cell walls removed, which is an extremely complex and time-consuming process that has not been well developed in most plants (Cunningham et al., 2018). The *Agrobacterium*-mediated method produces random DNA insertion, which probably can lead to the dysfunction of critical genes or introduction into genome positions with weak or unstable expression (Gelvin, 2017). In addition, the *Agrobacterium*-mediated method shows stronger regeneration and delivery effects in dicotyledonous plants (dicots) than in monocotyledonous plants (monocots) (Nyaboga et al., 2014) and has been utilized infrequently in chloroplast transformation. As the preferred chloroplast transformation method, biolistic particle delivery requires gold or tungsten particles combined with foreign DNA in order to penetrate the cell walls *via* bombardment, but this process typically causes extensive damage to plant tissues and results in low levels of gene expression in the process.

The challenges of classic delivery methods are characterized by their tissue specificity and narrow host range, even between individual cultivars from the same species (Nyaboga et al., 2014), and an effective method of target DNA transfer for chloroplast transformation is urgently needed to overcome the above-mentioned disadvantages. In recent years, nanotechnology has become a powerful tool in plant biotechnology by providing modular transport chassis applicable for the transportation of biomolecules, nanosensors, nanotherapeutics, and chemicals. Giraldo et al. have previously found that artificially manufactured nanoparticles, single-walled carbon nanotubes, can easily pass through the rigid plant cell walls, cell membranes, and even double lipid bilayers (outer and inner membrane) of chloroplasts smoothly (Giraldo et al., 2014). In addition, Kwak et al. used breakthroughs in the research of using nanomaterials as a carrier to deliver foreign DNA into plant chloroplasts (Kwak et al., 2019). They designed and screened single-walled carbon nanotubes to selectively transfer DNA into chloroplasts derived from various plants species without additional chemicals or biolistics. This nanoparticle-mediated delivery tool has practical advantages relative to classic delivery techniques and is regarded as a potential transformation method with high efficiency in plants.

A difficulty intrinsic with chloroplast genome transformation is attributed to the high polyploidy of the plastomes. In order to generate a stable transplastomic line, it is typically necessary to provide selective pressure until all copies of chloroplast DNA in the absence of transgenes are removed. Several rounds of selection are often essential, resulting in a longer transformation procedure than those associated with nuclear transgene processes. Recently, Jakubiec et al. had exploited a novel technique for foreign gene delivery and expression in chloroplasts. This technology differs from traditional methods because it does not need to integrate foreign transgenes into the chloroplast genome. The foreign gene is instead amplified as an individual unit named “minichromosome” in the chloroplast (Jakubiec et al., 2021). By identifying a specially appointed flanking sequence within the “minichromosome”, the helper protein can start the replication processes (Jakubiec et al., 2021).

## Application of CRISPR/Cas9 editing systems in chloroplast gene expression

Editing the genome within the plant has a tremendous potential to enhance crop yield and, in turn, to meet progressively larger agricultural and environmental challenges. As a rapidly developing RNA-guided genome editing tool, CRISPR-Cas9 has been widely studied and used in plant nuclear gene editing (Li et al., 2013; Nekrasov et al., 2013; Shan et al., 2013). However, the use of this tool is rarely reported in chloroplast gene editing, which is likely due to the difficulty of transporting both the Cas9 protein and the guide RNA to the chloroplasts and expressing these two critical elements in the chloroplasts at the same time (Kim et al., 2021).

It is widely recognized that some free-living photosynthetic cyanobacteria were entrapped by eukaryotic cells approximately 2 billion years ago, eventually giving rise to modern-day chloroplasts (Daniell et al., 2021). Therefore, chloroplast genome sequences share conserved properties with ancestral cyanobacteria. Researchers can use the similarity between chloroplast and cyanobacteria genomes to identify new methods of chloroplast gene editing, and this strategy has resulted in new attempts that have been made in microalgae in recent years—for example, the use of CRISPR/Cas9 editing in microalgae was first suggested by Jiang and his coworkers, but successful transformants were difficult to generate due to the potential toxicity of the constitutive expression of Cas9 protein in *Chlamydomonas reinhardtii* (Jiang et al., 2014). The CRISPR/Cas9 system has also been successfully applied to cyanobacterial genome editing for producing succinate *via* the deleted glucose-1-phosphate adenylyltransferase gene (Li et al., 2016). In 2020, researchers from the genome engineering company Napigen demonstrated a new CRISPR-mediated organelle genome editing technique called “Edit Plasmids” and successfully performed proof-of-concept experiments in *Chlamydomonas reinhardtii* chloroplasts (Yoo et al., 2020). The Edit plasmid can replicate in the mitochondria or chloroplasts independently. It consists of four parts: Cas9 expression box, guided RNA expression box, donor DNA, and optional markers. In addition, donor DNA does not carry functional gRNA target sites, so it is not resected by highly active Cas9/gRNAs. Unfortunately, problems such as improving the editing efficiency of this method, digesting unmodified organelle DNA, and promoting homogeneity remain as challenges.

At present, there is no CRISPR/Cas9 system suitable for chloroplast genomes in plants; however, researchers from South Korea have developed an efficient chloroplast editing technology (Kim et al., 2021). They designed a Golden Gate cloning system consisting of 424 transcription activator-like effector and 16 expression plasmids to produce DddA-derived Cytosine Base Editor (DdCBE) plasmids and then created a DdCBE system to increase the efficiency of point mutagenesis in chloroplasts. They used the DdCBEs to induce base editing with chloroplast genomes in lettuce and rapeseed calli and demonstrated editing frequency as high as 38%. In addition, they generated plantlets and lettuce calli with editing frequencies as high as 99%, which were resistant to spectinomycin and streptomycin. Further studies will focus on investigating whether heterogeneity induced by DdCBEs produce phenotypic effects and whether chloroplast genome editing ability can be strengthened by modifying the DdCBE system.

## Regulation of chloroplast gene expression using RNA-binding proteins

RNA plays several key roles in plant cells, including regulation of gene expression, gene information transfer, scaffold construction of macromolecular structures, and reaction catalyzation. RNA combines with RNA-binding proteins to form ribonucleoprotein complexes. The RNA-binding proteins manipulate various processes of RNA life activities (Glisovic et al., 2008). Thus, controlling or revising the properties of RNA by using artificially designed RNA-binding proteins is a farsighted approach in plant biotechnological applications. RNA manipulation may also be reversible and therefore more useful than DNA editing.

Among RNA-binding proteins, the best characterized are the pentatricopeptide repeat (PPR) proteins, which are particularly prevalent in terrestrial plants. Most PPR proteins mediate RNA to impact multiple aspects of metabolic processes within organelles (Barkan and Small, 2014). Although PPR proteins are encoded by nuclear genes, they almost exclusively target and function in the chloroplasts and mitochondria and are inherently highly sequence-specific (Gully et al., 2015). Therefore, many laboratories have designed artificial Designer Pentatricopeptide Repeat Proteins (dPPRs) to manipulate the RNA abundance. In 2014, Coquille et al. successfully designed synthetic PPR domains according to conserved residues within PPRs. The results indicated that the dPPR domains were highly soluble and bound targeted RNA in a controlled, sequence-specific pattern (Coquille et al., 2014). Two years later, to examine how dPPR proteins recognize and interact with their single-stranded RNA (ssRNA) targets specifically, Ping Yin’s group synthesized and expressed a series of artificial PPR proteins that can bind to specific sites of target ssRNAs. Their studies not only provided elaborate modular and specific binding models of dPPR repeats but also laid solid foundations for improving the RNA manipulation technology (Shen et al., 2016).

To date, few dPPR *in vivo* experiments have been successful. In 2019, Barkan’s laboratory expressed dPPR proteins from nuclear transgenes to induce an approximately 40-fold increase in the expression of plastid foreign genes, the maximal protein accumulation of which was close to Rubisco level (Rojas et al., 2019). In another experiment, Barkan’s laboratory successfully designed a dPPR protein in transgenic *Arabidopsis* plants and used it in genetic engineering research to bind a specific mRNA sequence in chloroplasts, demonstrating that the synthetic dPPR protein can reliably and selectively combine with targeted RNA *in vivo* (Mcdermott et al., 2019). In another case, Hammani et al. carried out a functional complementation experiment to show that synthetic dPPR protein binds to its expected mRNA target with specificity *in vivo* and successfully replaced a natural PPR protein by stably processing *rbcL* mRNA (Manavski et al., 2021). These results indicated that dPPR proteins can be artificially designed and modified to approximate the functions of natural PPR proteins and highlighted methods that can be used to regulate when, where, and to what extent chloroplast genes are expressed. The growing repertoire of dPPR proteins with clear RNA binding sites represents tools to exploit the unique properties of the chloroplast gene expression system.

## Regulation of chloroplast gene expression at the post-transcriptional level

Small artificial biomolecules such as aptamers may affect proteins (Gong et al., 2014a; Bao et al., 2017). Peptide aptamers are small peptides (approximately eight to 20 amino acids in length) that have a peptide loop in an inert scaffold protein and can specifically bind to target proteins. Peptide aptamers interact to disrupt protein function; thus, small peptides can act as powerful competitive inhibitors and can specifically interrupt and/or prevent the generation of protein–protein interactions by covering the original binding sites.

Peptide aptamers have been developed and successfully applied to reverse genetic approaches for the efficient and precise perturbation of protein function in plants (Song et al., 2013; Gong et al., 2014a)—for example, Song et al. designed and exploited an antagonistic peptide to functionally analyze CLV3/ESR-related family members in *Arabidopsis*. Researchers created a useful interference system and successfully disturbed CLV3 protein function in cultivated plants in a nutrient medium comprised of the artificial synthetic peptide (Song et al., 2013). To investigate the function of EJC core component MAGO–Y14 protein, Gong’s group introduced a peptide aptamer into rice (Gong et al., 2014a; Gong et al., 2014b). They identified a highly specific aptamer PAP containing a 16-amino-acid random peptide that antagonizes rice MAGO *via* yeast two-hybrid systems. The BiFC and Pulldown analyses showed that the rice MAGO protein effectively and specifically interacted with PAP through disrupting the MAGO–Y14 interaction (Gong et al., 2014a). Moreover, PAP transgenic plants exhibited more serious phenotypic defects than *MAGO* or *MAGO–Y14*RNAi plants (Gong et al., 2014a; Gong et al., 2014c), demonstrating that competitive conjunction or the interaction of PAP to MAGO can affect the heterodimer formation of natural MAGO–Y14 and impair its function in rice (Gong et al., 2014a).

In conclusion, recent research have shown that peptide aptamers can be used in a direct, substitutable, and compensatory method for plant functional genomics study. Peptide aptamers directly affect the function of protein or protein complexes, acting as a complementary method for other tools that play a part at the DNA and RNA levels. At present, few studies have been reported on the use of peptide aptamers in the plant research field, and most uses remain in the “proof-of-concept” stage. In the future, peptide aptamers may, for example, fused to chloroplast transport peptide structures which can cross the chloroplast membrane and interfere with the function of chloroplast-gene-encoded proteins.

## Summary and outlook

**Figure 1:**
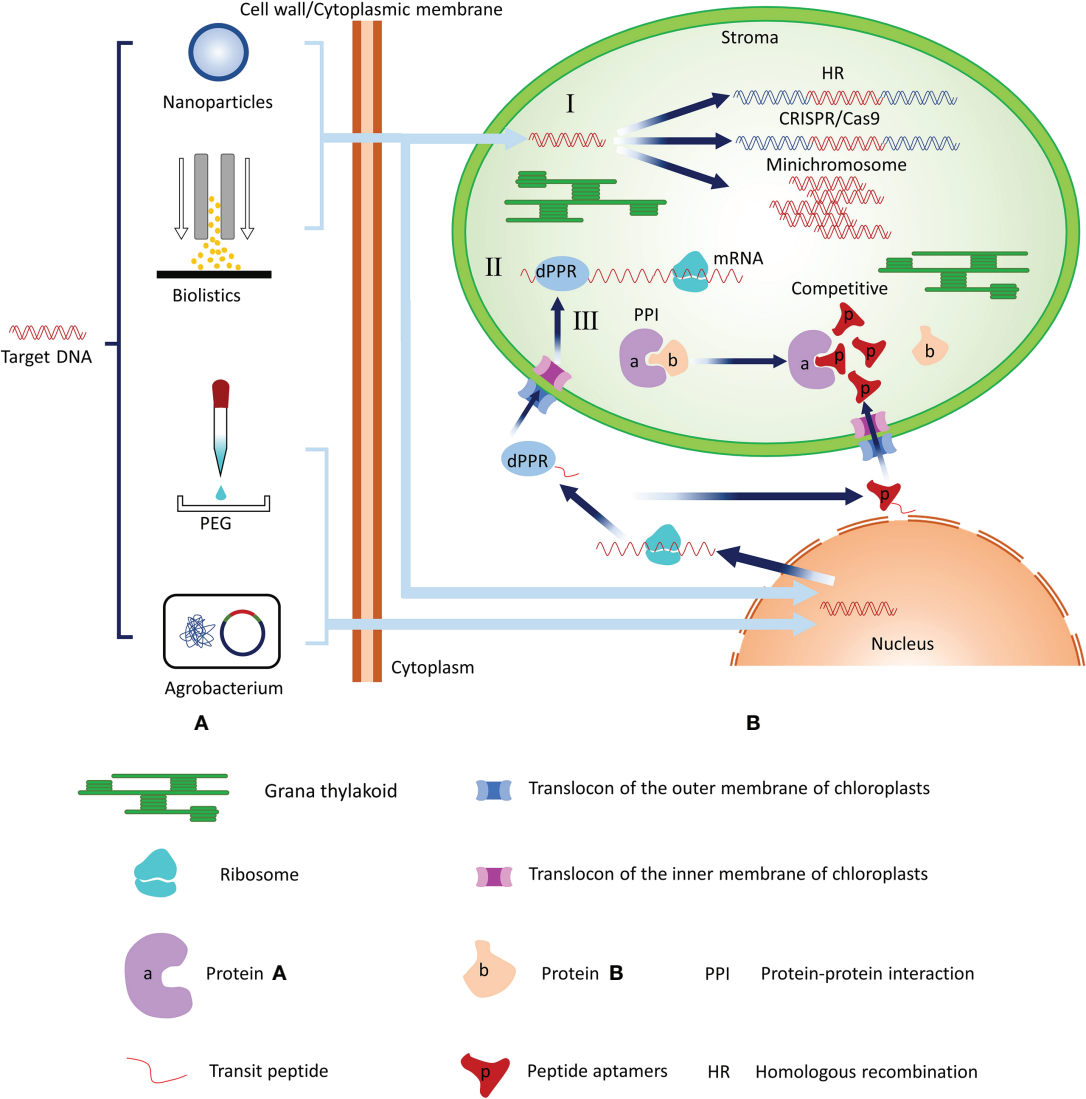
Summary of the chloroplast transformation technologies and potential regulation methods for chloroplast gene expression. **(A)** Technologies that introduce target genes into chloroplasts include biolistics and nanoparticle-mediated transformation. Biolistics and nanoparticle-mediated methods require that foreign plasmid DNA be adhered to the surface of metal particles or wrapped in nanoparticles and then DNA be mediated by power of free-power into chloroplasts or nucleus. PEG can change the structure of cell membranes such that foreign plasmid DNA can enter plant cells and nucleus. The *Agrobacterium* infection method transforms the recombinant plasmid into competent cells of *Agrobacterium* and then infect plant cells with *Agrobacterium* to achieve nuclear transformation. **(B)** I: Regulation of chloroplast gene expression at the DNA level. Target genes can directly enter the chloroplast and regulate gene expression at the DNA level through CRISPR/Cas9 techniques *via* the HR method or foreign DNA functions as an independent minichromosome. II: Regulation of chloroplast gene expression at the RNA level. After the *dPPR* gene enters the recipient cell, it replicates and expresses dPPR protein. The fused transit peptide of the dPPR protein guides it to enter the chloroplast through the TOC/TIC transport complex. TOC, translocase at the outer envelope membrane of the chloroplast; TIC, translocase at the inter-chloroplast membrane. The dPPR protein directly binds with mRNA to regulate the expression of chloroplast genes. III: Regulation of chloroplast gene expression. After the target gene enters the recipient cell, it replicates and expresses in the nucleus to construct the aptamer. The aptamer with fused transit peptide can be guided into the chloroplast and interacts with the corresponding chloroplast protein to affect the chloroplast protein function.

Chloroplasts (plastids) are the defining organelles of photosynthetic organisms. In addition to executing normal photosynthesis, chloroplasts play roles in many other metabolic pathways and provide access both in the engineering of intrinsic metabolic pathways and in the addition of new biochemical pathways. Therefore, chloroplasts are the biosynthetic factories in plant cells. In recent years, studies have expanded the toolbox that can be used for chloroplast genome engineering by a wide margin, and new systems to regulate the expression of plastid transgenes have been developed (Yoo et al., 2020; Jakubiec et al., 2021; Kim et al., 2021; Newkirk et al., 2021). In this review, we described and summarized chloroplast transformation methods and suggested potential regulation methods that may be applicable to chloroplast engineering in the future (**Figure 1**).

Designing an effective chloroplast transformation scheme for new species depends on the improvement of transformation systems, and the engineering of chloroplast genomes *via* genetic transformation is only currently implemented in a few species due to limited transformation systems (PEG-mediated transformation, *Agrobacterium*-mediated delivery, biolistics-mediated delivery, *etc*). Due to the lack of new transformation technology, the development of chloroplast engineering in different plant species is restricted (Bock, 2015). However, nanotechnology and minichromosome may be used to address the most difficult challenges in chloroplast biotechnology studies (Jakubiec et al., 2021; Newkirk et al., 2021). The interaction between nanoparticles and plant cells is an area of interest that requires further development, and the actual mechanism through which nanoparticles enter plant cells and cross chloroplast bilayer membranes has not been identified. With a well-developed understanding of the interactions between plants and nanoparticles, accurate nanoparticle-based biomolecule transport to chloroplasts in a variety of plant species may become possible. The study of minichromosome indicates that it is a powerful approach in chloroplast transgene expression and organelle genome engineering (Jakubiec et al., 2021). This method can effectively reduce the screening procedure by avoiding the polyploidy of the plastomes under selective pressure. Thus, the “minichromosome” tool is expected to make a broader impact on agricultural and industrial applications.

Plant chloroplast genomes encode a large number of genes necessary for photosynthesis or related metabolism. Approaches or tools for editing genes in chloroplasts are essential for exploring the functions of these genes, raising crop productivity, and improving crop traits. Burgeoning genome engineering technologies, particularly CRISPR–Cas9-based approaches, have been widely applied to knock out or change the gene function in nuclear DNA. In the future, newly modified CRISPR/Cas9-based approaches may be useful for chloroplast gene editing. PPR proteins with specific sequences can be artificially designed and used for RNA editing within chloroplasts in a predictable, sequence-specific manner *via* fusion with chloroplast transport peptides and encoded in the nucleus (**Figure 1**). The peptide aptamers method may reverse genetic strategies to precisely and efficiently disrupt plant protein function without compromising gene structure or expression (**Figure 1**). Thus, using new technologies and tools such as Crispr/CAS9, dPPR proteins, and aptamers to regulate chloroplast gene expression may enhance chloroplast engineering and increase the number of possible plant recipients (**Figure 1**). Finally, the breathtaking pace of advancement in chloroplast engineering will be not only conducive to generating plants traits that are valuable to humans but also helpful to improve the photosynthesis efficiency of chloroplasts.

## Author contributions

JX designed the outlines in this review. YA, YW, and XW wrote the original manuscript. JX edited the manuscript. All authors contributed to the article and approved the submitted version.

## Funding

This work was supported by the Fundamental Research Funds for the Central Universities (grant number 2021ZY58) and National Natural Science Foundation of China (grant number 32201516).

## Conflict of interest

The authors declare that the research was conducted in the absence of any commercial or financial relationships that could be construed as a potential conflict of interest.

## Publisher’s note

All claims expressed in this article are solely those of the authors and do not necessarily represent those of their affiliated organizations, or those of the publisher, the editors and the reviewers. Any product that may be evaluated in this article, or claim that may be made by its manufacturer, is not guaranteed or endorsed by the publisher.
